# Pain-Related Cognitive Processes, Pain Interference, and Alexithymia in Patients With Primary Headaches

**DOI:** 10.7759/cureus.39688

**Published:** 2023-05-30

**Authors:** İlteriş Ahmet Şentürk, Suna Aşkın Turan, Tuğba Eyigürbüz, Erman Şentürk, Nilüfer Kale İçen

**Affiliations:** 1 Pain Management, Bağcılar Education and Research Hospital, İstanbul, TUR; 2 Pain Management, Mersin City Education and Research Hospital, Mersin, TUR; 3 Neurology, Bağcılar Education and Research Hospital, İstanbul, TUR; 4 Psychiatry, NP Feneryolu Medical Center, Üsküdar University, İstanbul, TUR

**Keywords:** pain-related anxiety, pain-related disability, alexithymia, pain interference, headache

## Abstract

Objectives

This study aims to investigate the effects of pain-related cognitive processes (PRCPs) and emotional state on pain-related disability (PRD) and pain interference (difficulty in performing daily routines, difficulty in engaging in social activities [the enjoyment of life], and the impact on work and/or school performance) in patients with primary headaches (PHs).

Methodology

PRCPs were evaluated with the Pain Anxiety Symptom Scale-20 (PASS-20), Pain Catastrophizing Scale (PCS), and Pain Belief Questionnaire (PBQ). Anxiety, depression, and alexithymia were investigated to assess the emotional state. PRD was assessed by Headache Impact Test-6 (HIT-6). Health-related quality of life (HRQoL) was evaluated under three headings: daily activities (with Short Form-36 [SF-36] Question [Q] 22), social activities (with Graded Chronic Pain Scale-Revised [GCPS-R] Q 4), as well as the working ability (with GCPS-R Q 5). Two separate models were constructed to identify the factors influencing PRD and HRQoL in PHP: M1 to reveal the factors affecting PRD and M2 to determine the independent factors affecting pain interference. In both models, correlation analysis was applied first and the significant data were then evaluated with regression analysis.

Results

A total of 364 participants (74 healthy controls [HCs] and 290 PHPs) completed the study. In M1, the following domains were significantly associated with PRD: cognitive anxiety (β = 0.098; 95% confidence interval [CI] = 0.001-0.405; *P* = 0.049); helplessness (β = 0.107; 95% CI = 0.018-0.356; *P* = 0.031); alexithymia (β = 0.077; 95% CI = 0.005-0.116; *P* = 0.033); depression (β = 0.083; 95% CI = 0.014-0.011; *P* = 0.025). In M2, factors associated with impairment in daily activities for PHP were as follows: duration of pain, pain intensity, alexithymia, escape-avoidance response, psychological anxiety, anxiety, and poor sleep quality (*R* = 0.770; *R*^2^ = 0.588). The independent factors affecting social activities for PHP were pain intensity and pain-related anxiety (*R* = 0.90; *R*^2^ = 0.81). Independent risk factors that affected the ability to work for PHP were pain intensity, cognitive anxiety, escape-avoidance response, and pain anxiety (*R* = 0.90; *R*^2^ = 0.81).

Conclusions

This study highlights the importance of cognitive and emotional processes that help increase our understanding of the patient with PHs. This understanding may help to reduce disability and improve the quality of life in this population by helping to guide multidisciplinary treatment goals.

## Introduction

The prevalence of headache disorders among adults is estimated to be about 50%, and one-third or more of these adults have migraines. Headaches that last 15 days or more a month affect 1.7% to 4% of the world’s adult population [1]. The International Headache Society (IHS) classifies headaches into the following types: primary, secondary, and painful cranial neuropathies; other facial pains; and other headaches [2]. Because of their prevalence and effects on health-related quality of life (HRQoL), primary headache disorders (PHDs) such as migraines, tension-type headaches (TTHs), and trigeminal autonomic cephalgias (TACs) are significant for public health [3].

In recent years, research on the mechanisms of chronic pain has shifted beyond focusing solely on nociception toward a broader perspective that includes pain-related cognitive (PRC), motivational, and psychiatric factors and processes that may contribute to the development and maintenance of chronic pain. These factors have been demonstrated to play a significant role in headache-related disability and/or HRQoL [4-5]​​​​​​. Pain is a subjective perceptual phenomenon that affects cognitive functions, and cognitive factors influence pain expectation and assessment [6]. The cognitive process contributes directly to one’s thoughts and experiences regarding pain (e.g., the increased neural response to pain perception and pain anticipation associated with pain-related rumination) [7]. Patients’ intense expectations about pain and the repetition of negative processes caused by pain, fear of pain, or worry about the negative consequences of pain all contribute significantly to disability [8]. The fear-avoidance model explains the relationship between pain-related anxiety and impairment [9]. Pain catastrophizing, one of the cognitive variables, refers to beliefs about pain and is a repetitive way of thinking that is associated with decreased problem-solving ability and negative effects [10]. Catastrophizing is associated with increased disability and pain severity and is an important cognitive measure and prognostic indicator in patients with chronic pain. From this perspective, it can be said that catastrophizing is one of the primary objectives of cognitive behavioral therapy for patients with headaches [11-12]. Pain-related negative beliefs, such as being convinced of the permanence of pain and regarding pain as mysterious and unexplainable, make coping with the pain more difficult [13].

Specifically, migraineurs have higher levels of harm avoidance and persistence and lower levels of self-direction [12]. Patients with PHD frequently exhibit psychiatric comorbidities, with anxiety disorders and depression being the most prevalent. Psychiatric comorbidities are associated with poorer pain management outcomes [12-14]​​​​​​.

Alexithymia is a cognitive-emotional disorder that has been associated with somatosensory amplification, which is a tendency to focus on benign or neutral somatic sensations due to difficulty in experiencing and expressing emotions. It is thought that individuals with alexithymia misinterpret somatic sensations in favor of physical signs of illness, focusing on somatic manifestations of emotional arousal, and that alexithymia is associated with greater pain intensity and disability [15].

Evaluating the relationship between cognition and pain is critical for understanding chronic pain syndromes and their psychosocial effects, attaining therapeutic goals, and improving treatment outcomes [6]. People filter environmental stimuli based on the mental patterns formed by their experiences and formulate expectations accordingly, and their reactions and feelings are influenced by how events are perceived and interpreted rather than by the events themselves [16]. From this perspective, when a person has a headache, they experience emotional responses and form certain thoughts and/or images in their mind. The current study aims to determine whether there is a significant relationship between various factors such as sociodemographic data, pain characteristics (duration, frequency, intensity, etc.), pain-related cognitive process (PRCP - pain-related anxiety, catastrophic thoughts, and pain beliefs), emotional status (alexithymia, depression, and anxiety), and pain outcomes (pain disability and pain interfering with the quality of life [QoL]).

## Materials and methods

Ethics committee

Approval for the study was obtained from the Istanbul Bağcılar Training and Research Hospital Clinical Research Ethics Committee (2021.01.1.06.006).

Participants

For this prospective study, data were obtained from three outpatient clinics (pain medicine, headache polyclinic, and neurology) at two centers (Istanbul Bağcılar Training and Research Hospital and Istanbul Cam and Sakura City Hospital). Those who presented to the clinics with complaints of headaches for at least three months were invited to enroll in the study. The diagnosis of PH was per the International Classification of Headache Disorders, Third Edition (ICHD-3) beta [2] criteria, and psychiatric examination of the participants was conducted per the Diagnostic and Statistical Manual of Mental Disorders, Fifth Edition (DSM-5VR) criteria [17]. All participants provided their written informed consent. Exclusion criteria were as follows: confirmed cognitive and psychological dysfunction (from examination of telemedicine records and personal history), other systemic medical disorders associated with chronic pain (e.g., rheumatoid arthritis, fibromyalgia, and cancer-related pain), head and/or neck injury, a history of substance abuse, the use of antidepressants-anxiolytic drugs for headache prophylaxis, and usage of painkillers to relieve headaches more than 10 days a month (to exclude medication overuse headaches), below 18 years old or 75 years old or more. In addition, a healthy control (HC) group was formed in the study comprising adults in comparable age groups who did not experience headaches or who had a headache fewer than three times per month that did not require treatment. The same exclusion criteria were applied to the control group.

The following details were recorded: demographic information, pain characteristics, duration of pain in months, headache location, duration of the attack, the presence of aura, accompanying symptoms (nausea/vomiting, photophobia, or phonophobia), any prior treatment, and the number of days per month they took analgesics.

Scales

Pain Variables

Pain intensity: The Numeric Rating Scale (NRS) was applied. Participants were instructed to select an integer (between 0 and 100) that most accurately described the level of pain. Scores between 50 and 70 were regarded as moderate, and those between 80 and 100 were regarded as high.

Headache-related disability: Headache Impact Test-6 (HIT-6) is a six-item self-reported questionnaire used to assess headache-related disability. It assesses the impact that headaches have had on psychological, cognitive, occupational, and social functioning over the previous four weeks. Scores range from 36 to 78, and scores above 60 indicate that headache has a very serious impact on functioning [18].

Pain grading: Graded Chronic Pain Scale-Revised (GCPS-R) was used. The six-item scale is used to measure chronic pain, which is divided into three severity categories: mild (Grade 1), bothersome (Grade 2), and high impact (Grade 3). High-impact chronic pain (HICP) is characterized by persistent pain that interferes with work, social life, and self-care [19].

Pain-Related Cognitive Processes

Pain-related anxiety: Pain-related anxiety was analyzed with the Pain Anxiety Symptom Scale-20 (PASS-20). This scale evaluates four aspects of pain-related anxiety: cognitive anxiety, escape/avoidance actions, fear of pain, and physiological symptoms of anxiety. PASS-20 is graded on a six-point Likert-like scale, with 0 being *never* and 5 being *always*. Higher scores indicate greater pain anxiety [20]. In our study, we utilized a five-point Likert scale (0 [*never*] to 4 [*always*]) because it was simpler to comprehend.

Catastrophic thoughts. The Pain Catastrophizing Scale (PCS) was used to measure pain-related catastrophic thoughts. It consists of three subscales: helplessness (Qs 1-5 and 12), magnification (Qs 6, 7, and 13), and rumination (Qs 8-11). Using a five-point Likert scale, evaluations (0 = *not at all* to 4 = *always*) are made. It is rated from 0 to 52 points, and a higher score corresponds to a higher level of pain-related catastrophic thoughts [21].

Beliefs: The Pain Belief Questionnaire (PBQ) was utilized. This measure has two subgroups: organic beliefs and psychological beliefs. Organic beliefs evaluate the perceived source of pain as well as the perceived physical harm or physiological discomfort that threatens its control. Psychological beliefs analyze internal elements and emotions that influence pain experience. Higher scores indicate a worse perception of pain and more negative beliefs [22].

Emotional Processes

Depression: The Beck Depression Inventory (BDI) is a 21-item assessment of the severity of depression. Each item is graded between 0 and 3 on a four-point Likert-like scale. Higher scores indicate more severe depressive symptoms [23].

Anxiety: The Beck Anxiety Inventory (BAI) is a 21-item scale for measuring the degree of anxiety. Each item is graded from 0 to 3 on a four-point Likert-like scale [24].

Alexithymia: Toronto Alexithymia Scale-20 (TAS-20) has three subscales: difficulty identifying feelings (DIFs), difficulty describing feelings to other people (DDF), and a stimulus-bound, externally oriented thinking (EOT) style. Alexithymia is present if the overall TAS-20 score is greater than 60, and possible alexithymia is present if the total score is between 52 and 60 [25].

QoL and Pain Interference

Sleep quality: The Pittsburgh Sleep Quality Index (PSQI) is a questionnaire designed to measure sleep quality and disruptions. Scores on the PSQI vary from 0 to 21 points. A total score of 5 or higher is a sensitive and specific indicator of poor sleep quality [26].

HRQoL: The short form-36 (SF-36) was applied. It comprises eight subscales (physical function, pain, limitation due to physical problems, limitation due to emotional problems, emotional well-being, social function, energy fatigue, and general health perception). The scale provides not just a single total score but also individual scores for each of the eight subscales. Subscales assess HRQoL on a scale of 0 to 100, with 0 indicating poor and 100 indicating high HRQoL [27].

Pain interference: Pain interference was assessed in three categories: daily activities (SF-36 Q 22), social activities (GCPS-R Q 4), and working ability (GCPS-R Q 5).

Allodynia

Cutaneous allodynia: The 12-item Allodynia Symptom Checklist (ASC-12) is a tool used to assess cutaneous allodynia. ASC-12 scores range from 0 to 24, with 3 to 5 suggesting mild allodynia, 6 to 8 indicating moderate allodynia, and 9 and higher indicating severe allodynia [28]. 

**Figure 1:**
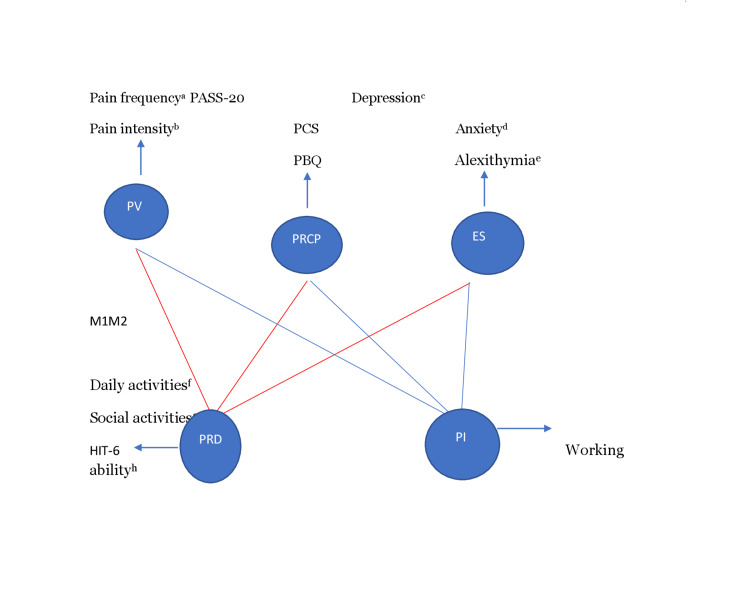
Diagram of scales used for variables. Figure credits: All the authors of this study. ^a^Pain frequency was calculated by the SF-36 Q 21. ^b^Pain intensity was calculated with NRS. ^c^Depression was calculated with BDI. ^d^Anxiety was calculated with BAI. ^e^Alexithymia was calculated with TAS-20. ^f^Daily activities were calculated by SF-36 Q 22. ^g^Social activities were calculated with GCPS-R Q 4. ^h^Working ability with GCPS-R Q 5. M1 (red lines): Correlation analysis pathway to reveal factors affecting PRD. (To determine the factors affecting PRD PV, PRCP and ES were first evaluated in the correlation analysis and then the results of the regression analysis were revealed after the significant data.) M2 (blue lines): Correlation analysis pathway to reveal PI (The steps applied in M1 were also applied in M2). TAS-20, Toronto Alexithymia Scale-20; BDI, Beck Depression Inventory; BAI, Beck Anxiety Inventory; Q, question; SF-36, Short Form-36; PV, pain variables; PRCP, pain-related cognitive process; PASS-20, Pain Anxiety Symptom Scale-20; PCS, Pain Catastrophizing Scale; PBQ, Pain Belief Questionnaire; ES, emotional state; PRD, pain-related disability; HIT-6, Headache Impact Test-6; PI, pain interference; M1, Model 1; M2, Model 2; NRS, Numeric Rating Scale; GCPS-R, Graded Chronic Pain Scale-Revised

In summary, standard questionnaires [29,30] were completed in this recommended order by the participants: pain frequency (SF-36 Q 21), pain intensity (NRS), pain disability (HIT-6), pain interference (SF-36, GCPS-R, and PSQI), PRCP (PASS-20, PCS, and PBQ), HRQoL (SF-36 and PSQI), emotional status (BDI, BAI, TAS-20, and SF-36), and allodynia (ASC-12) (Figure 1).

Hypothesis and models

We hypothesized that negative thoughts and/or images may affect current and future headache experiences, may evoke negative emotions, and may cause the individual to focus excessively on their physical limitations and pain experience, thereby increasing their pain perception. Based on this hypothesis, two models were devised: M1 to reveal factors affecting pain-related disability (PRD) and M2 to determine independent factors that affect pain interference were constructed. In both models, all variables were first evaluated by correlation analysis. Statistically significant data were then included in regression analysis. Multiple linear regression analysis was used to clarify the cause-and-effect relationship between the scales.

Statistical analysis

IBM SPSS version 23 (IBM Corp., Armonk, NY, USA) was used to conduct statistical analysis. The findings are presented as a sum of numbers (*N*) and percentages (%) or mean ± standard deviation (SD). The normality of numerical variables was examined using the Kolmogorov-Smirnov test. The Kruskal-Wallis test was used on non-normally distributed variables, and Bonferroni correction *M* was applied while performing the Mann-Whitney U test to determine the significance of pairwise differences. The percentages of categorical variables were compared between the groups by using the chi-square (*X*^2^) test, and post hoc analysis was done as necessary. The Mann-Whitney U test was performed to compare the variables of individuals with and without headaches.

The strength of the associations between the dependent variable and several independent variables was measured using the multiple regression coefficients (*R*). The multiple explanatory coefficients (*R*^2^) values indicate how well the independent variable explains the dependent variable, and this was required to be high for a model to be considered adequate. The *F* statistic had to be significant, and the standard error (SE) of the regression equation had to be modest for the model to be considered significant. When the standard error was high, *R*^2^ was tested to determine whether the model was adequate.

## Results

Comparison of demographic data and pain variables

Of the 364 participants who completed the study, 74 (20.3%) were HCs. In 290 PHPs, chronic migraine (CM) (76, or 20.8%) and episodic migraine without aura (EMWOA) (57, or 15.6%) were the most common diagnoses. Table 1 summarizes the comparisons between PHP and HCs in terms of demographic data and pain variables.

Demographic Data

There was a statistical difference between patients with primary trigeminal neuralgia (pTN) and the other groups in terms of mean age and age groups (*X*^2^ = 33.29; *P* < 0.001). The proportion of male participants was higher in the TAC group (*X*^2^ = 22.7; *P* = 0.002). In terms of employment, marital status, and the presence of chronic disease, there was a statistical difference between pTN patients and other groups (*P* < 0.050). There were no differences between the groups in other demographic data.

Pain Variables

Participants with headaches reported a mean pain duration of 74.79 ± 91.31 months of pain, and HCs reported a mean duration of 4.51 ± 10.92 months of pain (*Z* = −11,707; *P* < 0.001).

Pain disability and intensity: A long disease duration and high headache frequency were significantly associated with headache-related disability (*r* = 0.550; *P* < 0.001). The PHP group had a pain intensity on the NRS of 7.18 ± 2.06, and the HCs had a pain intensity of 1.71 ± 1.64 (*Z* = −12.538; *P* < 0.001). In the PHP, the calculation of PRD was 63.82 ± 7.57, and 121 (33.2%) participants reported having very severe headaches. As expected, the pain load was higher in PHPs compared to the control group.

Pain interference: Daily activities (SF-36 Q 22) scores were 47.32 ± 25.46 for the PHP group and 85.81 ± 20.30 for HCs (*Z* = −10.074; *P* < 0.001). The social activities (GCPS-R Q 4) score was 6.72 ± 2.74 in PHPs and 1.8 ± 2.25 in HCs (*Z* = −10.678; *P* < 0.001). Working ability (GCPS-R Q 5) scores were 6.63 ± 2.72 in the PHP group and 1.66 ± 1.98 in HCs (*Z* = −11.098; *P* < 0.001). Sleep quality scores were 8.28 ± 3.62 in the PHP group and 4.93 ± 2.39 in HCs (*Z* = −7.413; *P* < 0.001) (data not shown in Table 1).

**Table 1:** Demographic characteristics of the participants. Values are presented as mean ± standard deviation or number (%). ^a^BMI calculated using the formula kg/m^2^. ^b^The period from the beginning of the complaints to the present (months). ^c^Pain intensity was calculated by the NRS. ^d^Pain-related disability was calculated by HIT-6. ^e^Pain interference was evaluated under four headings: daily activities with SF-36 Q 22; social activities with GCPS-R Q 4; working ability with GCPS-R Q 5; sleep quality with PSQI. ^f^Allodynia was calculated by ASC-12. ASC-12, Allodynia Symptom Checklist-12; SF-36, Short Form-36; HC, healthy control; EMWA, episodic migraine with aura; EMWOA, episodic migraine without aura; CM, chronic migraine; ETTH, episodic tension-type headache; CTTH, chronic tension-type headache; pTN, primary trigeminal neuralgia; TOS, trigeminal autonomic cephalgia; BMI, body mass index; DM, diabetes mellitus; HT, hypertension; TD, thyroid dysfunction; CAD, coronary artery disease; NRS, numeric rating scale; HIT-6, Headache Impact Test-6; Q, question; GCPS-R, Graded Chronic Pain Scale-Revised; PSQI, Pittsburgh Sleep Quality Index

	HC (*n* = 74)	EMWA (*n* = 31)	EMWOA (*n* = 57)	CM (*n* = 76)	ETTH (*n* = 35)	CTTH (*n* = 41)	pTN (*n* = 21)	TOS (*n* = 29)	X^2^	P
Age (years)	38 ± 11.34	35.96 ± 10.35	36.91 ± 10.68	38.72 ± 13.03	33.80 ± 12.48	40.85 ± 11.63	58.90 ± 17.19	36.72 ± 11.05	33.296	0.001
Age range (years)										
18-35	30 (40.5%)	14 (45.2%)	21 (36.8%)	29 (38.2%)	19 (54.3%)	10 (24.4%)	3 (14.3%)	14 (48.3%)	39.106	0.001
35-50	34 (45.9%)	13 (41.9%)	30 (52.6%)	31 (40.8%)	12 (34.3%)	21 (51.2%)	4 (19%)	8 (27.6%)		
50-65	9 (12.2%)	4 (11.9%)	6 (10.5%)	14 (18.4%)	4 (11.4%)	8 (19.5%)	3 (14.3%)	7 (24.1%)		
≥65	1 (1.4%)	0 (0%)	0 (0%)	2 (2.6%)	0 (0%)	2 (4.9%)	11 (52.4%)	0 (0%)		
Gender										
Women	41 (55.4%)	22 (71%)	45 (78.9%)	60 (78.9%)	28 (80%)	29 (70.7%)	14 (66.7%)	13 (44.8%)	22.7	0.002
Men	33 (44.6%)	9 (29%)	12 (21.1%)	16 (21.1%)	7 (20%)	12 (29.3%)	7(33.3%)	16 (55.2%)		
BMI^a^	24.14 ± 4.41	25.63 ± 4.65	25.92 ± 5.43	24.65 ± 4.63	25.68 ± 5.25	26.91 ± 4.95	26.38 ± 5.67	24.13 ± 4.44	12.785	0.078
Working status										
Working	52 (70.3%)	12 (38.7%)	28 (49.1%)	28 (36.8%)	15 (42.9%)	17 (41.5%)	3 (14.3%)	19 (65.5%)	109.607	0.001
Left job	3 (4.1%)	6 (19.4%)	9 (15.8%)	9 (11.8%)	4 (11.4%)	11 (26.8%)	3 (14.3%)	2 (6.9%)		
Never	10 (13.5%)	11 (35.5%)	17 (29.8%)	27 (35.5%)	14 (40%)	10 (24.4%)	6 (28.6%)	3 (10.3%)		
Retired	3 (4.1%)	1 (3.2%)	1 (1.8%)	4 (5.3%)	0 (0%)	2 (4.9%)	9 (42.9%)	4 (13.8%)		
Student	6 (8.1%)	1 (3.2%)	2 (3.5%)	8 (10.5%)	2 (5.7%)	1 (2.4%)	0 (0%)	1 (3.4%)		
Education level										
Course	3 (4.1%)	2 (6.5%)	3 (5.3%)	3 (3.9%)	1 (2.9%)	3 (7.3%)	4 (19%)	0 (0%)	50.642	0.055
Primary	8 (10.8%)	8 (25.8%)	18 (31.6%)	20 (26.3%)	10 (28.6%)	11 (26.8%)	5 (23.8%)	3 (10.3%)		
Middle school	7 (9.5%)	5 (16.1%)	6 (10.5%)	11 (14.5%)	4 (11.4%)	7 (17.1%)	4 (19%)	1 (3.4%)		
High school	17 (23%)	8 (25.8%)	13 (22.8%)	26 (34.2%)	11 (31.4%)	13 (31.7%)	4 (19%)	14 (48.3%)		
University	39 (52.7)	8 (25.8)	17 (29.8%)	16 (21.1)	9 (25.7)	7 (17.1)	4 (19%)	11 (37.9%)		
Marital status										
Single	24 (32.4%)	10 (32.3%)	15 (26.3%)	20 (26.3%)	14 (40%)	9 (22%)	1 (4.8%)	14 (48.3%)	51.152	0.005
Married	44 (59.5%)	21 (67.7%)	37 (64.9%)	51 (67.1%)	18 (51.4%)	27 (65.9%)	16 (76.2%)	15 (51.7%)		
Widow	1 (1.4%)	0 (0%)	2 (3.5%)	3 (3.9%)	1 (2.9%)	3 (7.3%)	4 (19%)	0 (0%)		
Divorced	5 (6.8%)	0 (0%)	2 (3.5%)	2 (2.6%)	0 (0%)	2 (4.9%)	0 (0%)	0 (0%)		
Living together	0 (0%)	0 (0%)	1 (1.8%)	0 (0%)	2 (5.7%)	0 (0%)	0 (0%)	0 (0%)		
Chronic diseases										
None	57 (77%)	17 (54.8%)	35 (61.4%)	44 (57.9%)	25 (71.4%)	27 (65.9%)	8 (38.1%)	22 (75.9%)	105.391	0.001
DM	5 (6.8%)	3 (9.7%)	1 (1.8%)	2 (2.6%)	3 (8.6%)	4 (9.8%)	2 (9.5%)	0 (0)		
HT	5 (6.8%)	1 (3.2%)	1 (1.8%)	8 (10.5%)	2 (5.7%)	1 (2.4%)	9 (42.9%)	2 (6.9%)		
TD	0 (0%)	1 (3.2%)	9 (15.8%)	4 (5.3%)	3 (8.6%)	1 (2.4%)	1 (4.8%)	1 (3.4%)		
CAD	2 (2.7%)	0 (0%)	2 (3.5%)	4 (5.3%)	1 (2.9%)	1 (2.4%)	0 (0%)	0 (0%)		
Asthma	1 (1.4%)	2 (6.5%)	6 (10.5%)	6 (7.9%)	0 (0%)	2 (4.9%)	1 (4.8%)	1 (3.4%)		
COPD	0 (0%)	0 (0%)	1 (1.8%)	2 (2.6%)	0 (0%)	1 (2.4%)	0 (0%)	2 (6.9%)		
Others	4 (5.4)	7 (22.6)	2 (3.5)	6 (7.9)	1 (2.9)	4 (9.8)	0 (0)	1 (3.4)		
Smoking (Yes)	36 (48.6%)	11 (35.5%)	11 (19.3%)	23 (30.3%)	9 (25.7%)	16 (39%)	9 (42.9%)	12 (41.4%)	15.717	0.028
Pain duration^b^	4.51 ± 10.92	90.00 ± 93.10	98.86 ± 105.67	95.52 ± 105.53	46.08 ± 57.59	60.77 ± 94.38	44.76 ± 55.34	37.37 ± 37.63	148.741	0.001
Pain intensity^c^ (Total)	1.71 ± 1.64	7.70 ± 1.14	6.45 ± 2.08	7.92 ± 1.75	5.25 ± 2.13	7.54 ± 1.65	8.04 ± 1.90	7.62 ± 2.19	194.274	0.001
Pain disability^d^ (Total)	42.12 ± 7.93	64.60 ± 6.33	64.94 ± 5.43	66.13 ± 8.10	60.51 ± 6.39	65.20 ± 9.26	61.47 ± 5.78	59.44 ± 7.85	172.093	0.001
Pain interference^e^										
Daily activities	85.81 ± 20.30	39.16 ± 23.38	52.63 ± 20.42	42.75 ± 24.78	58.57 ± 24.95	51.42 ± 24.95	46.42 ± 26.55	42.24 ± 30.69	115.077	0.001
Social activities	1.83 ± 2.25	7.16 ± 1.78	6.07 ± 2.72	7.21 ± 2.73	5.05 ± 2.42	7.14 ± 2.57	7.33 ± 3.41	8.00 ± 2.03	135.077	0.001
Working ability	1.66 ± 1.98	7.60 ± 1.71	5.75 ± 2.80	7.07 ± 2.56	4.85 ± 2.85	6.97 ± 2.53	7.66 ± 2.61	7.62 ± 2.36	147.886	0.001
Sleep quality	4.93 ± 2.39	7.80 ± 2.70	7.80 ± 3.54	8.71 ± 3.74	7.02 ± 3.13	8.00 ± 3.91	8.76 ± 3.92	9.75 ± 3.95	65.500	0.001
Allodynia^f^ (Total)	1.23 ± 2.67	3.06 ± 4.11	2.17 ± 3.00	3.65 ± 4.66	0.91 ± 1.59	1.42 ± 2.03	2.47 ± 3.98	1.79 ± 3.21	28.593	0.001

Comparison of PRCPs and emotional status

Table 2 summarizes the relationship between PRCPs and emotional status.

**Table 2:** Assessment of the cognitive process. Values are presented as mean ± standard deviation. ^a^Pain-related catastrophizing thoughts were calculated with PCS. ^b^Pain-related anxiety was calculated with PASS-20. ^c^Pain beliefs were calculated with PBQ. ^d^Anxiety was calculated with BAI. ^e^Depression was calculated with BDI. ^f^Alexithymia was calculated with TAS-20. ^g^Mental health was calculated with SF-36. ^h^Emotional role difficulty was calculated with the SF-36. TAS-20, Toronto Alexithymia Scale-20; BDI, Beck Depression Inventory; BAI, Beck Anxiety Inventory; PBQ, Pain Beliefs Questionnaire; SF-36, Short Form-36; PASS-20, Pain Anxiety Symptom Scale-20; PCS, Pain Catastrophizing Scale; HC, healthy control; EMWA, episodic migraine with aura; EMWOA, episodic migraine without aura; CM, chronic migraine; ETTH, episodic tension-type headache; CTTH, chronic tension-type headache; pTN, primary trigeminal neuralgia; TOS, trigeminal autonomic cephalgia

	HC (*n* = 74)	EMWA (*n* = 31)	EMWOA (*n* = 57)	CM (*n* = 76)	ETTH (*n* = 35)	CTTH (*n* = 41)	pTN (*n* = 21)	TOS (*n* = 29)	*X*^2^	*P*
Pain-related catastrophizing thoughts^a^										
Rumination	3.36 ± 3.53	9.41 ± 4.35	8.91 ± 4.55	10.43 ± 4.45	6.34 ± 4.60	9.80 ± 4.32	11.66 ± 2.74	9.89 ± 4.26	107.764	0.001
Magnification	2.16 ± 2.04	5.54 ± 2.71	5.45 ± 2.98	6.55 ± 2.85	3.62 ± 2.52	5.14 ± 2.83	7.57 ± 1.83	6.34 ± 2.68	115.056	0.001
Helplessness	3.97 ± 3.51	11.38 ± 5.59	11.31 ± 5.81	14.14 ± 6.22	7.88 ± 6.02	12.14 ± 6.46	14.85 ± 4.09	13.55 ± 5.85	126.305	0.001
Total	9.50 ± 8.14	26.35 ± 11.16	25.68 ± 12.23	31.13 ± 12.40	17.85 ± 11.91	27.09 ± 12.31	34.09 ± 6.92	29.79 ± 10.78	134.044	0.001
Pain-related anxiety^b^										
Cognitive anxiety	5.00 ± 4.34	12.38 ± 4.22	12.28 ± 4.50	12.80 ± 4.82	9.37 ± 4.86	12.36 ± 4.65	13.66 ± 5.14	13.44 ± 5.25	107.179	0.001
Fear	3.25 ± 3.47	8.83 ± 4.49	8.82 ± 4.79	10.38 ± 4.83	6.22 ± 4.08	9.56 ± 4.07	13.61 ± 4.87	12.79 ± 5.12	127.933	0.001
Physiological anxiety	1.55 ± 2.39	7.96 ± 4.42	6.10 ± 5.12	8.65 ± 5.34	3.97 ± 3.76	6.07 ± 4.60	11.85 ± 5.08	10.31 ± 6.11	128.181	0.001
Escape avoidance	6.54 ± 4.76	12.38 ± 4.46	12.17 ± 3.71	11.80 ± 4.84	9.37 ± 4.79	11.56 ± 4.48	14.38 ± 4.00	13.24 ± 4.34	78.665	0.001
Total	16.35 ± 12.30	41.58 ± 13.26	39.38 ± 13.77	43.64 ± 15.00	28.94 ± 14.12	39.56 ± 13.31	53.52 ± 15.57	49.79 ± 17.23	145.693	0.001
Pain beliefs^c^										
Organic beliefs	25.32 ± 7.01	21.03 ± 4.58	21.86 ± 6.20	20.35 ± 6.27	23.80 ± 5.94	20.29 ± 6.22	18.42 ± 4.63	19.24 ± 4.86	40.672	0.001
Psychogenic beliefs	9.75 ± 4.85	9.16 ± 3.05	8.36 ± 3.90	8.46 ± 3.80	9.28 ± 3.82	9.31 ± 3.74	8.61 ± 3.20	9.58 ± 3.31	6.385	0.496
Anxiety^d^ (Total)	8.64 ± 6.26	22.12 ± 14.24	18.03 ± 12.75	23.69 ± 14.20	14.94 ± 9.36	18.46 ± 12.97	19.04 ± 14.18	17.41 ± 10.19	60.602	0.001
Depression^e^ (Total)	9.14 ± 5.40	16.61 ± 10.47	13.49 ± 10.06	20.36 ± 11.14	16.54 ± 12.37	17.80 ± 8.80	25.09 ± 12.31	23.10 ± 11.04	71.620	0.001
Alexithymia^f^										
Difficulty describing feelings	10.41 ± 3.79	12.12 ± 3.94	11.94 ± 3.40	13.06 ± 4.18	11.42 ± 3.55	12.36 ± 3.20	10.00 ± 2.60	9.34 ± 3.07	35.546	0.001
Difficulty identifying feelings	12.95 ± 5.44	16.48 ± 6.75	14.84 ± 5.60	18.07 ± 6.76	14.82 ± 6.11	17.39 ± 6.32	12.71 ± 3.93	13.13 ± 4.96	39.830	0.001
Externally oriented thinking	19.45 ± 8.22	19.96 ± 6.17	19.45 ± 6.88	22.30 ± 6.58	21.11 ± 6.18	20.56 ± 5.70	16.09 ± 5.12	15.20 ± 4.78	33.030	0.001
Total	42.83 ± 15.46	48.58 ± 14.56	46.24 ± 13.45	53.44 ± 15.43	47.37 ± 13.13	50.31 ± 12.91	38.81 ± 9.85	37.69 ± 11.95	41.949	0.001
Mental health^g^	66.64 ± 19.00	45.41 ± 13.56	50.59 ± 19.67	46.71 ± 17.82	49.37 ± 18.76	48.58 ± 18.40	47.81 ± 17.36	45.51 ± 13.62	56.139	0.001
Emotional role difficulty^h^	74.77 ± 36.93	44.08 ± 44.21	49.70 ± 39.40	39.47 ± 39.14	60.95 ± 39.17	50.44 ± 42.84	41.36 ± 42.02	36.78 ± 36.01	37.455	0.001

Pain-Related Cognitive Processes

Catastrophic thoughts: In this category, the helplessness score was 12.24 ± 6.21 in the PHP group and 3.97 ± 3.51 in HCs (*Z* = −9.904; *P* < 0.001). The PCS total scores were 27.45 ± 12.38 in the PHP group and 9.50 ± 8.14 in HCs (*Z* = −10.143; *P* < 0.001).

Pain-related anxiety: Cognitive anxiety was scored as 12.30 ± 4.84 in the PHP group and 5.00 ± 4.34 in HCs (*Z* = −9.640; *P* < 0.001). The score for fear of pain was 9.76 ± 4.99 in the PHP group and 3.2 (SD = 3.47) in HCs (*Z* = −9.575; *P* < 0.001). The total pain-related anxiety scores for PHP were 41.56 ± 15.68 and 16.35 ± 12.35 for HCs (*Z* = −10.549; *P* < 0.001).

Pain-related beliefs: There was a significant difference between the group with PH and HCs with respect to pain-related organic beliefs (*Z* = −5.09; *P* < 0.001). However, there was no significant difference in terms of psychogenic beliefs between the groups (*P* = 0.393).

Emotional Status

As expected, scores for general anxiety and depression were greater in the PHP group than in HCs. In terms of anxiety, the PHP group scored 19.65 ± 13.09 and the HCs group scored 8.64 ± 6.26 (*Z* = −7.019; *P* < 0.001). For depression, the PHP group scored 18.40 ± 11.24, while the HCs group scored 9.14 ± 5.40 (*Z* = −6.622; *P* < 0.001). Differences were observed in the evaluation of alexithymia between PHP and HCs: for DDF, between CM and HCs (*X*^2^ = 35.546; *P* < 0.050 by post hoc tests); for DIF (all the above acronyms not previously defined), between CM and HCs (*X*^2^ = 39.830; *P* < 0.050 by post hoc tests); for EOT, between TACs and HCs (*X*^2^ = 33.030; *P* < 0.050 by post hoc tests); and total scores, between CM and HCs (*X*^2^ = 41.949; *P* < 0.050 by post hoc tests). There was no significant difference in the EOT subscale for the assessment of alexithymia between the PHP and HCs groups (*P* = 0.902). There was a significant difference between PHP and HCs for mental health (SF-36; *Z* = −7.286; *P* < 0.001), while for emotional role difficulty, there was a significant difference between HCs and EMWA, HCs and CM, and HCs and TAC patients (*P* < 0.050 with post hoc tests).

Hypothesis and models

Some data such as age, pain frequency, attack duration, and pain beliefs were not included in the models as only data that were significant in the preliminary correlation analysis were used in the regression modeling.

Model 1

The regression model of M1 was generated using the stepwise technique with the data that were significant in the correlation analysis, and M1 was significant (*F* = 4.47; *P* = 0.350; D-W value = 1.99). In the results of the regression analysis, a significant positive correlation was found in the variables indicated for PRD: BMI (β = 0.079; 95% confidence interval [CI] = 0.026-0.341; *P* = 0.022), pain duration (β = 0.126; 95% CI = 0.008-0.027; *P* < 0.001), pain intensity (β = 0.366; 95% CI = 1.039-1.832; *P* < 0.001), cognitive anxiety (β = 0.098; 95% CI = 0.001-0.405; *P* = 0.049), helplessness (β = 0.107; 95% CI = 0.018-0.356; *P* = 0.031), alexithymia (total) (β = 0.077; 95% CI = 0.005-0.116; *P* = 0.033), depression (β = 0.083; 95% CI = 0.014-0.011; *P* = 0.025), and sleep latency (β = 0.084; 95% CI = 0.185-1.824; *P* = 0.016). A negative connection was observed between general health perception and PRD (β = −0.083; 95% CI = −0.089 to −0.003; *P* = 0.035; *R* = 0.80) (Table 3).

**Table 3:** The effects of sociodemographic data, pain characteristics, pain-related cognitive processes, and alexithymia on pain-related disability. ^a^BMI calculated using the formula kg/m^2^. ^b^The period from the beginning of the complaints to the present (months). ^c^Pain intensity was calculated by NRS. ^d^Cognitive anxiety is a subscale of PASS-20. ^e^Helplessness is a subscale of PCS. ^f^Alexithymia was calculated with TAS-20. ^g^Depression was calculated with BDI. ^h^General health was calculated with SF-36. ^i^Sleep latency was calculated with PSQI. NRS, Numeric Rating Scale; TAS-20, Toronto Alexithymia Scale-20; BDI, Beck Depression Inventory; SF-36, Short Form-36; PASS-20, Pain Anxiety Symptom Scale-20; PCS, Pain Catastrophizing Scale; PSQI, Pittsburgh Sleep Quality Index; CI, confidence interval; β, beta; BMI, body mass index

	Unstandardized coefficients		Standardized coefficients	t	P	95% CI for *B*	
	B	SE	β			Lower bound	Upper bound
Constant	39.171	2.253		17.383	0.001	34.739	43.603
BMI^a^	0.184	0.080	0.079	2.293	0.022	0.026	0.341
Pain duration^b^	0.017	0.005	0.126	3.650	0.001	0.008	0.027
Pain intensity^c^	1.435	0.202	0.366	7.117	0.001	1.039	1.832
Cognitive anxiety^d^	0.203	0.103	0.098	1.975	0.049	0.001	0.405
Helplessness^e^	0.187	0.086	0.107	2.171	0.031	0.018	0.356
Alexithymie^f^ (total)	0.060	0.028	0.077	2.146	0.033	0.005	0.116
Depression^g^	0.088	0.039	0.083	2.252	0.025	0.164	0.011
General health^h^	–0.046	0.022	–0.083	–2.114	0.035	–0.089	–0.003
Sleep latency^i^	1.004	0.417	0.084	2.411	0.016	0.185	1.824

Model 2

The same procedure was followed for pain interference, and a regression analysis model was created for M2. Simple linear regression analysis results for social activities were significant (*F* = 4.57; *P* = 0.033; D-W value = 2.15).

Social activities: Between pain intensity and social activities (β = 0.208; 95% CI = 0.143-0.321; *P* < 0.001) and between pain-related anxiety (total) and social activities (β = 0.066; 95% CI = 0.001-0.023; *P* = 0.033), there were significant positive associations (*R* = 0.90; *R*^2^ = 0.81).

Working ability: Regression analysis results for workability were significant (*F* = 4.95; *P* = 0.027; D-W value = 1.86). Positive correlations for workability were observed for the following: pain intensity (β = 0.154; 95% CI = 0.083-0.259; *P* < 0.001), cognitive anxiety (β = 0.091; 95% CI = 0.001-0.107; *P* = 0.048), escape-avoidance response (β = 0.100; 95% CI = 0.010-0.119; *P* = 0.019), and pain anxiety (total) (β = 0.127; 95% CI = 0.012-0.034; *P* < 0.001). There was a negative correlation between social functioning and workability (β = −0.056; 95% CI = −0.014 to −0.001; *P* = 0.027; *R* = 0.90; *R*^2^ = 0.81).

Daily activities: Regression analysis model results within daily activities were significant (*F* = 4.52; *P* = 0.004; D-W value = 2.10). Significant negative correlations for daily activities were found for the following: pain duration (β = −0.098; 95% CI = −0.060 to −0.010; *P* = 0.006), pain intensity (β = −0.165; 95% CI = −0.845 to −2.564; *P* < 0.001 ), alexithymia (β = −0.085; 95% CI = −0.332 to −0.021; *P* = 0.026), escape-avoidance response (β = −0.204; 95% CI = −1.709 to −0.738; *P* < 0.001), psychological anxiety (β = −0.186; 95% CI = −0.523 to −1.543; *P* = < 0.001), anxiety (β = −.130; 95% CI = −0.515 to −0.106; *P* = 0.003), and sleep quality (β = −0.100; 95% CI = −6.077 to −1.258; *P* = 0.003; *R* = 0.770; *R*^2^ = 0.588) (Table 4).

**Table 4:** Relationships between sociodemographic data, pain characteristics, pain-related cognitive processes and, emotional state with pain interference. ^a^Pain intensity was calculated with NRS. ^b^Pain anxiety was calculated with PASS-20. ^c^Cognitive anxiety is a subscale of PASS-20. ^d^Escape is a subscale of PASS-20. ^e^Social functioning was calculated with SF-36. ^f^The period from the beginning of the complaints to the present (months). ^g^Alexithymia was calculated with TAS-20. ^h^Psychological anxiety is a subscale of PASS-20. ^i^Anxiety was calculated with BAI. ^j^Sleep quality with PSQI. NRS, Numeric Rating Scale; TAS-20, Toronto Alexithymia Scale-20; BAI, Beck Anxiety Inventory; SF-36, Short Form-36; PASS-20, Pain Anxiety Symptom Scale-20; PCS, Pain Catastrophizing Scale; PSQI, Pittsburgh Sleep Quality Index; CI, confidence interval; β, beta

	Unstandardized coefficients		Standardized coefficients	t	P	95% CI for *B*	
	B	SE	β			Lower Bound	Upper Bound
Social activities							
Constant	–0.124	0.188		–0.659	0.510	–0.494	0.246
Pain intensity^a^	0.232	0.045	0.208	5.110	0.001	0.143	0.321
Pain anxiety^b^	0.012	0.006	0.066	2.139	0.033	0.001	0.023
Working ability							
Constant	0.309	0.335		0.923	0.357	–0.349	0.967
Pain intensity	0.171	0.045	0.154	3.812	0.001	0.083	0.259
Cognitive anxiety^c^	0.054	0.027	0.091	1.983	0.048	0	0.107
Pain anxiety (total)	0.023	0.006	0.127	4.117	0.001	0.012	0.034
Escape^d^	0.065	0.027	0.100	2.348	0.019	0.010	0.119
Social functioning^e^	–0.007	0.003	–0.056	–2.224	0.027	–0.014	–0.001
Daily activities							
Constant	7.462	6.538		1.141	0.255	–5.396	20.321
Pain duration^f^	–0.035	0.013	–0.098	–2.747	0.006	–0.060	–0.010
Pain intensity	–1.704	0.437	–0.165	–3.901	0.001	–0.845	–2.564
Alexithymie^g^ (total)	–0.177	0.079	–0.085	–2.235	0.026	–0.332	–0.021
Escape	–1.224	0.247	–0.204	–4.958	0.001	–1.709	–0.738
Psychological anxiety^h^	–1.033	0.259	–0.186	3.986	0.001	–0.523	–1.543
Anxiety^i^	–0.310	0.104	–0.130	–2.989	0.003	–0.515	–0.106
Sleep quality^j^	–3.668	1.225	–0.100	–2.994	0.003	–6.077	–1.258

## Discussion

This prospective study, conducted in headache and pain outpatient clinics in two distinct centers in Turkey, analyzed pain characteristics, PRCPs, and emotional status in PHPs in comparison to the control group and determined the effects of these variables on PRD and HRQoL.

Comparison of patients with PHs and HCs

As predicted [31], the mean age of patients with pTN was higher than that of other groups; therefore, the proportion of retirees, the marriage rate, and the presence of comorbidities differed between those with pTN and other groups. Again, the ratio of men to women was greater in the TAC group. Other sociodemographic parameters did not show significant differences between other diagnostic categories, which may be a result of the relative homogeneity of these groups. There was a statistically significant difference between the PHP and the control group in terms of pain characteristics (duration, frequency, intensity, and disability), PRCP (all subscales and total scores except psychogenic pain beliefs), emotional status (depression, anxiety, and alexithymia), and HRQoL. These findings support earlier research [32-36]. Interestingly, in our study, we found a difference between patients with TACs and HCs for EOT, while there was only a difference between patients with CM and the control group for subscales of alexithymia other than EOT (DDF and DIF) and alexithymia total scores. Recent research has found that the effects of the EOT subscale were either less pronounced or non-existent compared to those of other alexithymia subscales [15]. The authors hypothesized that this was due to the poor psychometric qualities of the EOT, particularly for non-Western cultures. In addition, they suggested that chronic pain and DIF may be more closely related. We were unable to draw any comparisons because there is no study in the literature analyzing EOT in a group with TACs. Our opinion is that more research is required to assess scales for the traits of alexithymia in PHPs.

Determination of factors affecting PRD and pain interference in patients with PHs

We developed two separate models (M1 and M2). HIT-6 was used to assess headache-related disability for M1. HIT-6 assesses the likelihood that social functioning, role function, vitality, cognitive functioning, and psychological distress are characteristics that may influence headache frequency and intensity [18,37].  Pain interference is a measurement of the degree to which pain interferes with physical, cognitive, emotional, and recreational activities, as well as enjoyment of life [38]. In this context, the dependent variables for M2 are daily activities, social activities, and working ability.

Previous studies have found different results for BMI and headache disability, emphasizing that associations should be supported by larger studies [39-40]. We found only BMI as an independent risk factor for disability among sociodemographic data in M1. Pain behavior, disability, and general psychological distress may increase as pain progresses from the acute phase to the chronic phase. On the other hand, adjustment to pain and disability may occur over time [41]. There are conflicting results in the literature regarding relationships between pain duration (story) and PRD and pain interference [42-43]. We found pain duration to be an independent risk factor for PRD in M1 and daily activities in M2. The effects of high pain intensity on disability and pain experience were investigated, and its possible contribution to pain management was emphasized because baseline pain intensity is amenable to intervention [44-45]. ^ ^Increased pain intensity is associated with increased healthcare burden and costs, in addition to worsening HRQoL [46]. The primary goal of pain management is to increase physical function while reducing pain [36]. In our regression models (M1 and M2), pain intensity was one of the strongest predictors of PRD and pain interference. We draw attention to basic pain intensity as it is manageable (measures and treatment methods). Sleep disturbance is common in PHPs, and there are associations between poor sleep quality and pain disability [47]. We found a relationship between sleep latency and disability. Sleep latency is the technical term for the length of time it takes to fall asleep. Sleep latency was found to be normal or longer in migraine patients compared to the normal population [48]. It would be appropriate to compare our data in further studies with larger samples.

Those who attribute more meaning to their pain and have intense anxiety about their pain may show higher levels of disability. Such thought processes can be modified in psychotherapeutic interventions [49]. The fear-avoidance model explains how pain-related anxiety contributes to disability [9]. Pain-related anxiety symptoms are more consistent for disability in patients with chronic pain than general anxiety symptoms [50]. In this study, we showed that not general anxiety but cognitive anxiety (difficulty concentrating on other things), which is one of the PASS-20 subscales, contributes to the development of headache-related disability and is associated with the ability to work. Cognitive anxiety was shown to be the most important determinant of the fear-response pattern in patients with chronic low back pain [51]. The authors suggested that the cognitive anxiety scale could help identify patients who may benefit from exposure treatment for back pain. Cognitive processes encompass the thoughts, beliefs, attributions, and attitudes someone may experience. Cognitions influence whether a patient engages in behaviors that reduce the likelihood of a headache attack, treatment adherence, how they cope with a headache attack, and consequently headache-related disability [32].^ ^The fact that cognitive anxiety resulting from headache-related cognitive distortions is the most important predictor of headache-related disability in this study reinforces the importance of restructuring unrealistic beliefs in routine treatment. Cognitive distortions about headache management and treatment can be replaced by more realistic beliefs with cognitive restructuring, and then possible maladaptive behaviors are reorganized. Thus, individuals would be preoccupied with learning more adaptive ways of managing headaches rather than with unattainable goals like eradicating such beliefs. Behavioral therapy is suggested to be as effective as pharmacological treatments, not only for the treatment of headaches but also for maintaining a lifelong response to headache treatment, and it is effective in reducing anxiety sensitivity and decreasing anxiety related to pain sensations [52]. Especially in migraineurs, catastrophic thoughts appear to be an indicator of dysfunction and lower quality of life, independent of other psychological variables (such as anxiety and depression) [12]. We identified helplessness from catastrophizing thoughts as one indicator of PRD. Helplessness reflects the inability to cope with pain, and its important (compared to other subscales of the PCS) role between pain and disability levels has been emphasized [44,53]. Another interesting finding in our study is that alexithymia is more effective than anxiety for headache-related disability. The coexistence of chronic pain and depression is common; alexithymia is also significantly associated with depression and may predispose to depression, and depression may mediate chronic pain and alexithymia [54]. ^ ^Although we observed that alexithymia and depression are risk factors for PRD, whether depression mediates alexithymia was not evaluated in this study.

Pain-related anxiety contributes significantly to the functional limitations of those affected by headaches [55]. Here, we established a link between pain-related anxiety and pain interferences. It has been reported that patients with chronic pain, which significantly affects their daily activities, tend to reduce their participation in exercises and therapies, and avoidance behavior should be targeted in treatment planning [56]. Also, a pathway from anxiety sensitivity and headache severity to fear of pain and further into avoidance and escape behavior was demonstrated in patients with headaches [55]. We established relationships between escape-avoidance responses and pain interference. We also detected a relationship between physiological anxiety (PASS-20 subscales) and daily activities. Physiological anxiety is the subscale most associated with sleep problems [57].^ ^Sleep disturbance is associated with more severe headaches and pain interventions [47].^ ^We did not establish a relationship between catastrophizing thoughts and pain interference as we did for pain-related anxiety. Our results should be supported by larger studies.

Several limitations exist in this study. The use of a self-administered questionnaire can result in questions being misunderstood, increasing the possibility of subjective responses. However, the evaluation of the sample by a neurologist and a psychiatrist contributed to the validation of the screening results. In addition, prophylactic drugs can directly affect the emotional state and outcomes. Patients who received prophylactic treatment were not included in the study, leading to the possibility of selection bias. Again, selection bias may occur with the invitation method to enroll subjects from clinics. Finally, generalization of outcomes may not be appropriate for the elderly; therefore, caution is recommended in this age group.

## Conclusions

We found the effects of PRCP in PHP on disability and HRQoL to be significant. We emphasize that PRCPs should be considered in treatment goals, such as the identification and management of anxiety and depression. Our findings assist in the multidisciplinary evaluation and treatment of the needs of patients affected by PHs.
